# Exploring cognitive and emotional symptoms associated with hippocampal subfield atrophy in drug-induced Parkinsonism

**DOI:** 10.3389/fnagi.2025.1566785

**Published:** 2025-07-09

**Authors:** Wei Zhou, MengYue Tang, Bo Cheng, Ling Sun, HongYu Lin, Yang Fan, Nian Liu, Shushan Zhang

**Affiliations:** ^1^Department of Neurology, Affiliated Hospital of North Sichuan Medical College, Nanchong, Sichuan, China; ^2^Sichuan Key Laboratory of Medical Imaging, Department of Radiology, Affiliated Hospital of North Sichuan Medical College, Nanchong, Sichuan, China; ^3^Department of Geriatrics, Nanchong Central Hospital, Nanchong, Sichuan, China

**Keywords:** Drug-induced Parkinsonism, Parkinson’s disease, hippocampus, subfields, cognitive impairment

## Abstract

**Background:**

Drug-induced Parkinsonism (DIP) is a secondary Parkinsonism with limited research on its hippocampal structural changes. This study explores hippocampal subfield volumes in DIP compared to Parkinson’s disease (PD) and healthy controls (HCs), investigating correlations with cognitive (Montreal Cognitive Assessment, MoCA), emotional (Hamilton Depression Rating Scale, HAMD; Hamilton Anxiety Rating Scale, HAMA), and motor (Unified Parkinson’s Disease Rating Scale, UPDRS) symptoms.

**Methods:**

A total of 19 DIP patients, 20 PD patients, and 20 HCs were enrolled. MRI-based hippocampal subfield volumes were assessed using FreeSurfer, and clinical scores were evaluated for cognitive, emotional, and motor functions. Statistical analyses compared group differences and examined correlations.

**Results:**

Significant atrophy was observed in the DIP group in multiple hippocampal subfields compared to HCs, including the presubiculum, subiculum, Granule cell and molecular layer of the dentate gyrus (GC-ML-DG), molecular_layer_HP, Cornu ammonis (CA) 1, CA4, hippocampal tail, and fimbria. MoCA scores positively correlated with volumes in bilateral hippocampus and subfields such as subiculum and CA4, while HAMD scores mainly showed negative correlations in both DIP and PD group. UPDRS scores revealed group-specific patterns, with DIP showing stronger associations between non-motor symptoms and hippocampal volume.

**Conclusion:**

This study first reported significant hippocampal subfield atrophy in DIP, distinct from PD, and links structural changes to cognitive, emotional, and motor impairments. These findings advance understanding of DIP pathophysiology and underscore the hippocampus’s role in non-motor symptoms.

## Introduction

Drug-induced parkinsonism (DIP) is one of the most common forms of secondary parkinsonism (Bondon-Guitton et al., 2011; Savica et al., 2017; Shiraiwa et al., 2018), resulting from the use of medications that block dopamine receptors or deplete dopamine levels (Feldman et al., 2022; Margolesky, 2019), and its prevalence and incidence of DIP increased in the recent years (Han et al., 2019). Although DIP and Parkinson’s disease (PD) are both subtypes of parkinsonism (Shin and Chung, 2012; Wenning et al., 2011), and DIP shares several clinical features with PD, such as bradykinesia and rigidity, the underlying neurobiological mechanisms of DIP remain poorly understood, with limited research focusing on its structural and functional brain changes. Unlike PD, which has been extensively studied, DIP has received far less attention, leaving significant gaps in our understanding of its neuropathological basis.

Neuroimaging offers valuable insights for diagnosing DIP, particularly in cases with clinical presentations that closely resemble PD (Pitton Rissardo and Caprara, 2023). Current MRI studies on DIP remain limited, with existing research primarily focusing on alterations in the substantia nigra (Sung et al., 2016) and white matter (Lee et al., 2017). Our previous study found structural (volume) alterations in the subcortical nuclei of DIP patients (Zhou et al., 2024). These studies underscore the scarcity of neuroimaging investigations into DIP, highlighting a need for further exploration to better understand its pathophysiology and distinguish it from PD.

The hippocampus serves as a critical brain region involved in cognitive processes and emotional regulation (Li et al., 2020; Zhang et al., 2016). Moreover, the hippocampus is divided into several substructures, each with distinct functions and vulnerabilities in neurodegenerative diseases. Its substructures, such as the dentate gyrus and CA regions, have distinct roles in memory, learning, and mood regulation and are known to be affected in neurodegenerative and psychiatric disorders. Previous studies have reported that hippocampal alteration is associated with cognitive function in PD patients (Yildiz et al., 2015), indicating its potential as a biomarker for disease progression and treatment response. Beyer et al. (2013) found that memory deficits in recall and recognition have been linked to hippocampal atrophy in newly diagnosed PD patients, particularly in verbal memory tasks. Furthermore, Low et al. (2019) have reported atrophy in specific hippocampal subfields, such as CA1, in individuals who progressed to PD dementia. These findings underscore the strong association between hippocampal dysfunction and cognitive impairment in PD. Cognitive impairment and emotional disturbances can also occur in patients with DIP. However, it remains unclear whether these changes are associated with hippocampal structural alterations in DIP patients, and to date, no studies have specifically investigated the relationship between hippocampal subfield volumes and clinical symptoms, such as cognitive deficits, depressive symptoms, and motor dysfunction, in this population.

Hence, our study is the first to explore the relationship between hippocampal subfield volumes and cognitive and emotional functioning in DIP patients, potentially offering new insights into the pathophysiology of DIP and its management.

This research not only provides new perspectives on the hippocampal structural alterations associated with DIP, but also helps us understand the potential relationship between these changes and cognitive, emotional, and motor symptoms.

## Methods

### Participants

The research protocol received approval from the Local Ethical Committee (Approval no. 2021ER0105-1), and all participants signed written informed consent forms.

The study involved participants who were part of a previous cohort study (Zhou et al., 2024). A total of 19 patients with DIP, 20 patients diagnosed with PD, and 20 healthy control participants (HCs) were enrolled. The diagnosis of PD was made according to the 2016 Chinese diagnostic guidelines. DIP cases were confirmed based on the following criteria: (1) exhibiting parkinsonism symptoms; (2) absence of prior parkinsonism before exposure to causative drugs; (3) symptom manifestation following drug usage; and (4) being right-handed. Exclusion criteria for DIP participants included: (1) a diagnosis of primary Parkinson’s disease or other identifiable causes of parkinsonism; (2) MRI contraindications (e.g., claustrophobia or presence of metallic implants); (3) structural brain damage or motion artifacts on MRI; (4) history of neurological disorders (e.g., stroke, head injury); or (5) unwillingness to participate.

For PD participants, inclusion criteria were: (1) diagnosis per the 2016 Chinese PD criteria; (2) voluntary consent; and (3) right-handedness. Exclusion criteria included: (1) secondary or atypical parkinsonism; (2) inability to cooperate with symptom evaluations; (3) MRI contraindications; (4) significant structural abnormalities or motion artifacts on MRI; and (5) unwillingness to participate.

HCs were age-and sex-matched, with all participants being right-handed. The exclusion criteria for HCs included: (1) psychiatric or neurological disorders; (2) MRI contraindications; (3) significant structural abnormalities or motion artifacts on MRI; and (4) unwillingness to participate.

The evaluation of clinical symptoms was conducted using the Unified Parkinson’s Disease Rating Scale (UPDRS) (Goetz et al., 2008) and the Hoehn-Yahr (H-Y) staging scale (Goetz et al., 2004). The assessment of motor symptoms was carried out with the UPDRS-III and H-Y staging scale, whereas the evaluation of non-motor symptoms and daily living experiences related to motor functions was performed using the UPDRS-I and UPDRS-II, respectively. Cognitive assessment was conducted using the Montreal Cognitive Assessment (MoCA). Additionally, the patients’ emotional state was evaluated through the Hamilton Depression Rating Scale (HAMD) and the Hamilton Anxiety Rating Scale (HAMA).

### MRI scan

MRI data were collected using a 3.0 T scanner (GE Discovery MR750, USA) equipped with a 32-channel head coil. High-resolution 3D-T1-weighted imaging was performed with the following parameters: repetition time (TR) of 8.3 ms, echo time (TE) of 3.3 ms, flip angle of 15°, field of view (FOV) of 240 × 240 mm, image matrix of 240 × 240, and slice thickness of 1.0 mm with no interslice gap.

### Imaging analysis

**Figure 1:**
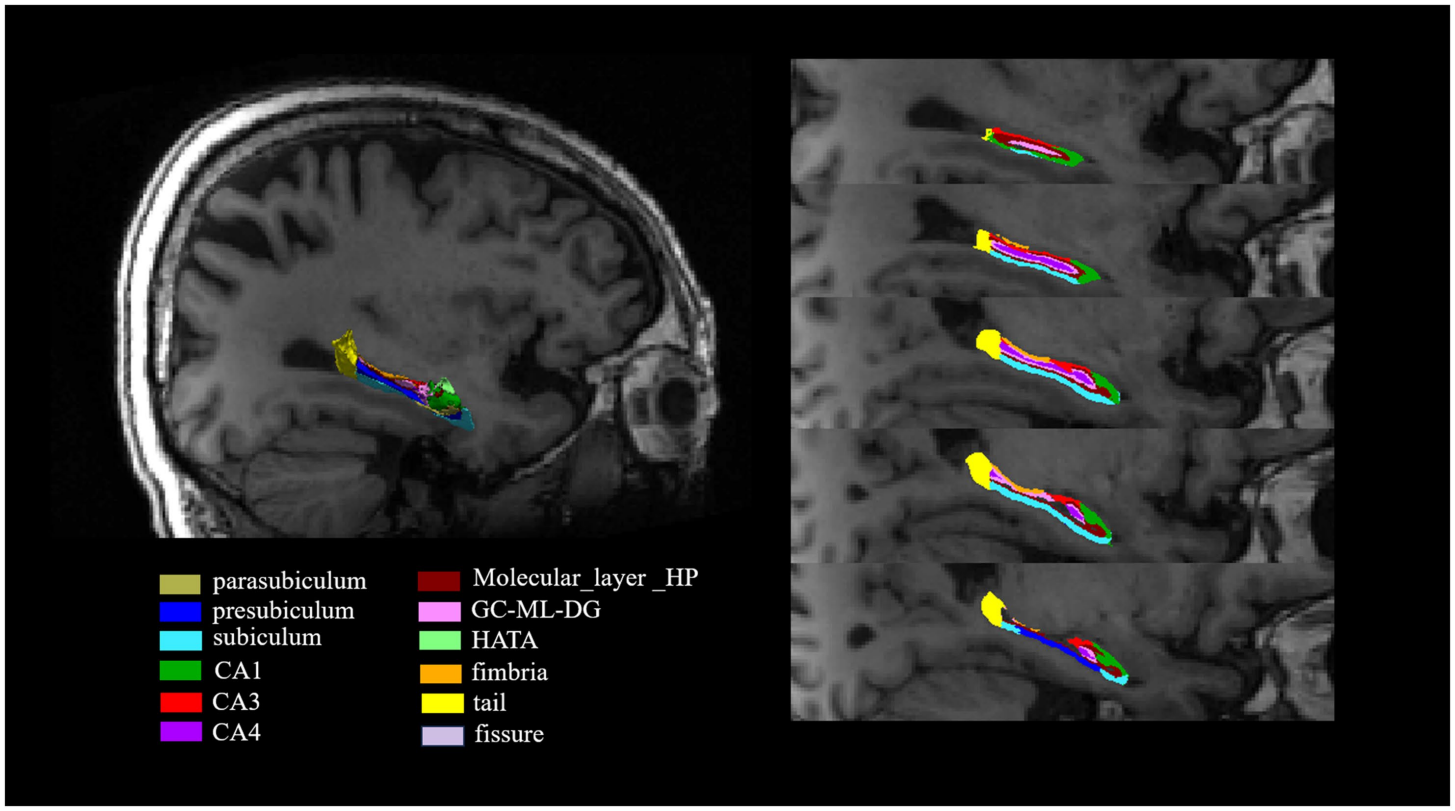
The segmentation of hippocampal subfields on T1-weighted MRI images. The hippocampus was segmented into the following subfields: parasubiculum, presubiculum, subiculum, CA1, CA3, CA4, molecular_layer_HP, GC-ML-DG, HATA, fimbria, tail and fissure. CA, Cornu ammonis; GC-ML-DG, Granule cell and molecular layer of the dentate gyrus; HATA, Hippocampus-amygdala transition area.

The hippocampal subfields segmentation was performed using FreeSurfer version 7.1.1. This automated process included several steps: correcting motion artifacts in T1-weighted images, aligning images to the Talairach coordinate system, adjusting for B1 field inhomogeneities, and applying a hybrid watershed algorithm for skull stripping. Subsequent stages involved labeling volumes, segmenting subcortical regions, refining subcortical structures, and constructing cortical models. The analysis specifically extracted volumes of 12 hippocampal subfields per hemisphere, including the Cornu ammonis (CA) 1, CA3, CA4, etc. (Figure 1). Additionally, the intracranial volume (ICV) was calculated. This hippocampal subfield segmentation approach has been widely used in neuroimaging research (Iglesias et al., 2015; Sämann et al., 2022).

### Statistical analysis

Continuous variables were expressed as either the mean or median. For H-Y staging scale, stages 2 and above were combined into a single group due to the limited number of patients in the higher stages (Ziegler et al., 2013). Comparisons of demographic and clinical features were conducted using analysis of variance (ANOVA), Mann–Whitney U-tests, or Chi-Squared tests. To examine hippocampal volume parameters among three groups, analysis of covariance (ANCOVA) was applied, followed by *post-hoc* analyses. *p*-values were adjusted for multiple comparisons using the false discovery rate (FDR). The relationship between clinical parameters and subcortical volume was explored through partial correlation analysis, controlling for sex, age, and ICV.

All statistical analyses were carried out with SPSS software (Version 23.0), with *p* < 0.05 considered statistically significant.

## Results

### Demographics

The demographic and clinical characteristics of these subject groups are showed in the Table 1. There were no significant differences in age, sex distribution among the three groups. The duration of illness was marginally longer in the PD group than in the DIP group, though this difference was not statistically significant. Cognitive performance, assessed using the MoCA score, showed a significant group difference, with the DIP group scoring lower than the PD group. Scores on the UPDRS subscales (I, II, and III) showed no significant differences among groups. Emotional states, evaluated using the HAMA and HAMD score, were similar across groups, with no statistically significant differences (*p* > 0.05).

**Table 1:** Demographic and clinical characteristics of all participants.

Characteristics	PD	DIP	HC	f/t/x^2^/z	*p*
*n*	20	19	20	–	–
Sex M/F	6/14	4/15	10/10	3.849	0.146
Age, mean ± SD	64.60 ± 7.63	63.47 ± 8.91	60.40 ± 6.61	1.570	0.217
Illness duration (years), median (range)	1 (0.1 ~ 4)	0.6 (0.1 ~ 2.6)	–	−1.947	0.051
MoCA, mean ± SD	19.60 ± 5.75	15.42 ± 5.46	–	2.342	0.026
UPDRS I, mean ± SD	9.85 ± 6.16	10.789 ± 7.58	–	−0.426	0.673
UPDRS II, mean ± SD	9.00 ± 4.24	7.631 ± 6.73	–	0.461	0.453
UPDRS III, mean ± SD	15.45 ± 6.57	11.89 ± 6.35	–	1.718	0.094
HAMA, mean ± SD	10.30 ± 4.94	12.11 ± 7.13	–	−0.923	0.362
HAMD, mean ± SD	12.70 ± 5.14	13.11 ± 3.71	–	−0.281	0.780
H-Y grade, N					
Grade 1	12	6		3.167	0.075
Grade ≥2	8	13			

PD, Parkinson’s disease; DIP, Drug-induced parkinsonism; HC, Healthy control; UPDRS, Unified Parkinson’s Disease Rating Scale; HAMD, Hamilton Depression Rating Scale; HAMA, Hamilton Anxiety Rating Scale.

### Comparison of hippocampal and subfield volumes

**Figure 2:**
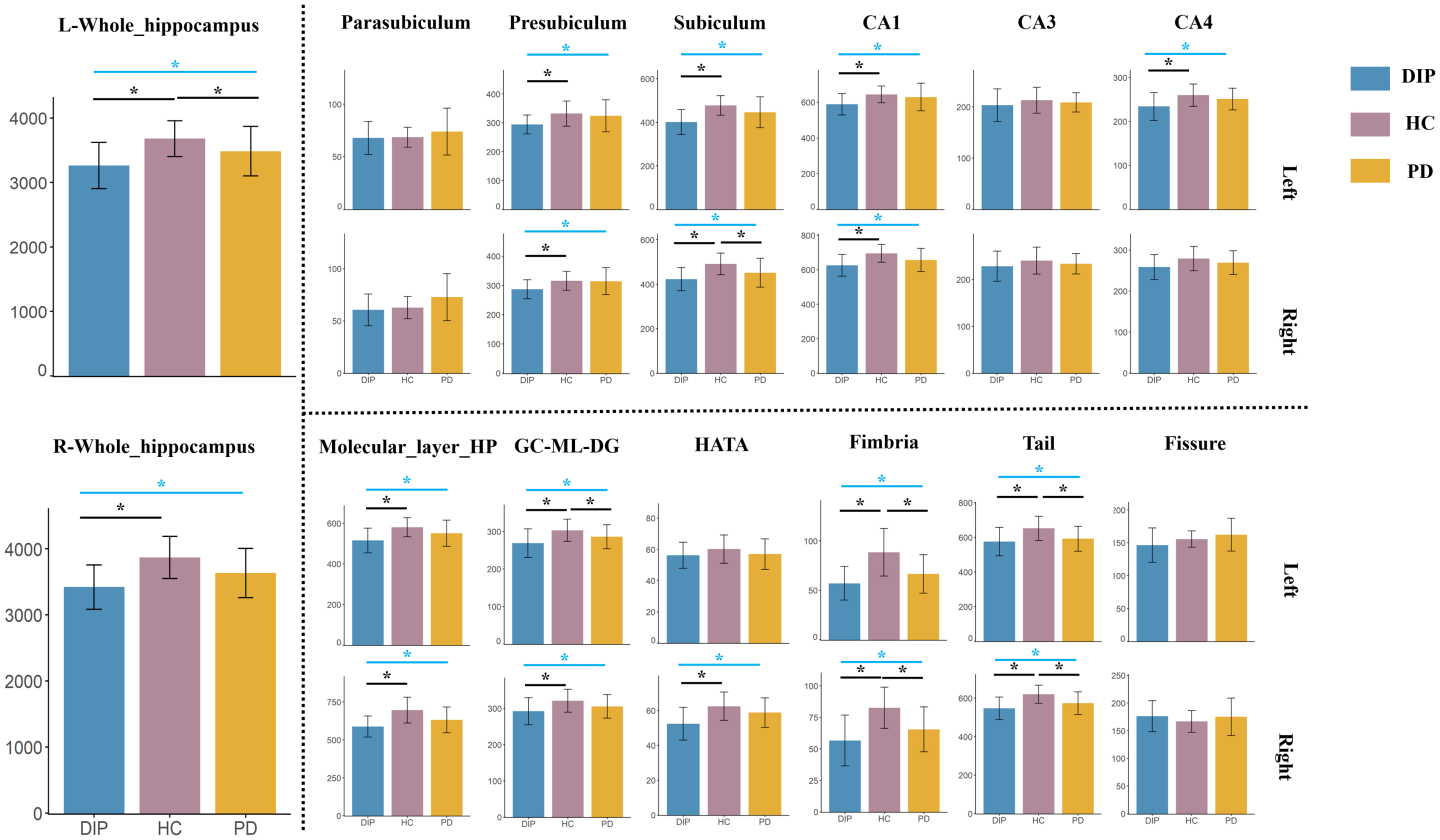
Comparison of Hippocampal Subfield Volumes among DIP, HC, and PD. The blue lines indicate the results of ANCOVA among these three groups, while the black lines represent *post-hoc* analysis. *Represents statistically significant differences (*p* < 0.05) in either the ANOVA or *post-hoc* comparisons.

The results revealed significant differences in hippocampal subfield volumes across the three groups, with the DIP group showing widespread reductions compared to HCs. Both the bilateral whole hippocampal volumes were significantly smaller in the DIP group, alongside reductions in several subfields, including the presubiculum, subiculum, Granule cell and molecular layer of the dentate gyrus (GC-ML-DG), Molecular_layer_HP, CA1, CA4, hippocampal tail, and fimbria. These reductions were bilateral and were more pronounced in DIP compared to HC, with some subfields also differing from the PD group. Overall, the DIP group exhibited the more severe hippocampal atrophy among the groups studied (Table 2 and Figure 2).

**Table 2:** Comparison of hippocampal and subfield volumes among the DIP, PD and HCs.

Hippocampus/Hippocampal subfields	PD		DIP		HC		*F-*value	*p*-value	P.fdr	PD vs. DIP	PD vs. HC	DIP vs. HC
	Mean	SD	Mean	SD	Mean	SD						
L_Whole_hippocampus	3485.959	385.228	3261.666	359.095	3679.575	279.442	9.449	0.000	0.000*	0.097	0.041*	0.000*
L_parasubiculum	73.881	22.334	67.846	15.711	68.540	9.457	1.224	0.302	0.329	–	–	–
L_presubiculum	324.380	55.629	294.330	32.923	332.292	43.366	4.760	0.013	0.020*	0.081	0.434	0.038*
L_subiculum	446.130	71.111	400.933	56.843	477.459	44.711	9.974	0.000	0.000*	0.052	0.058	0.001*
L_CA1	629.233	77.869	588.794	60.242	644.187	47.073	5.553	0.006	0.012*	0.175	0.237	0.003*
L_CA3	208.788	18.867	203.337	31.838	213.101	25.282	0.796	0.456	0.456	–	–	–
L_CA4	251.138	24.892	234.245	31.918	259.862	25.423	6.438	0.003	0.007*	0.113	0.136	0.003*
L_molecular_layer_HP	550.540	64.481	514.973	60.888	580.297	47.106	7.422	0.001	0.003*	0.135	0.076	0.002*
L_GC-ML-DG	285.889	31.899	268.815	38.063	303.125	29.522	7.826	0.001	0.003*	0.185	0.033*	0.001*
L_HATA	56.998	9.807	56.212	8.401	60.201	9.038	3.492	0.038	0.051	–	–	–
L_fimbria	66.627	19.459	56.977	16.978	88.415	24.118	7.797	0.001	0.003*	0.098	0.032*	0.004*
L_Hippocampal_tail	592.354	72.326	575.204	82.157	652.095	69.393	5.134	0.009	0.015*	0.425	0.037*	0.003*
L_hippocampal-fissure	162.361	25.008	146.394	26.243	155.661	12.506	2.077	0.135	0.162	–	–	–
R_Whole_hippocampus	3633.677	373.401	3419.987	335.977	3867.794	317.239	8.695	0.001	0.001*	0.082	0.081	0.000*
R_parasubiculum	72.763	22.543	60.568	15.202	62.718	10.671	1.926	0.156	0.187	–	–	–
R_presubiculum	314.535	46.613	286.962	32.306	315.894	32.380	4.956	0.011	0.019*	0.094	0.427	0.004*
R_subiculum	451.420	64.976	422.907	52.023	491.559	48.661	7.703	0.001	0.006*	0.183	0.045*	0.001*
R_CA1	656.910	67.770	625.945	62.959	695.484	51.441	6.139	0.004	0.012*	0.220	0.142	0.001*
R_CA3	233.554	21.833	228.278	32.133	240.219	28.800	1.255	0.293	0.320	–	–	–
R_CA4	269.070	28.985	258.567	30.599	279.162	29.416	3.445	0.039	0.052	–	–	–
R_molecular_layer_HP	631.983	85.152	588.281	69.610	696.656	84.586	5.874	0.005	0.012*	0.068	0.164	0.001*
R_GC-ML-DG	305.614	32.500	292.230	37.571	321.179	31.622	4.665	0.014	0.021*	0.320	0.113	0.002*
HATA	58.795	8.472	52.394	9.419	62.414	8.108	5.174	0.009	0.018*	0.053	0.698	0.003*
R_fimbria	65.493	17.810	56.687	20.129	82.596	16.324	6.408	0.003	0.012*	0.094	0.027*	0.004*
R_Hippocampal_tail	573.539	59.158	547.168	58.328	619.915	46.827	9.242	0.000	0.000*	0.183	0.021*	0.000*
R_hippocampal-fissure	175.076	33.815	176.197	28.152	166.776	19.667	0.187	0.830	0.830	–	–	–

PD, Parkinson’s disease; DIP, Drug-induced parkinsonism; HC, Healthy control. CA, Cornu ammonis; GC-ML-DG, Granule cell and molecular layer of the dentate gyrus; HATA, Hippocampus-amygdala transition area.*p < 0.05.

### Correlation analysis

**Figure 3:**
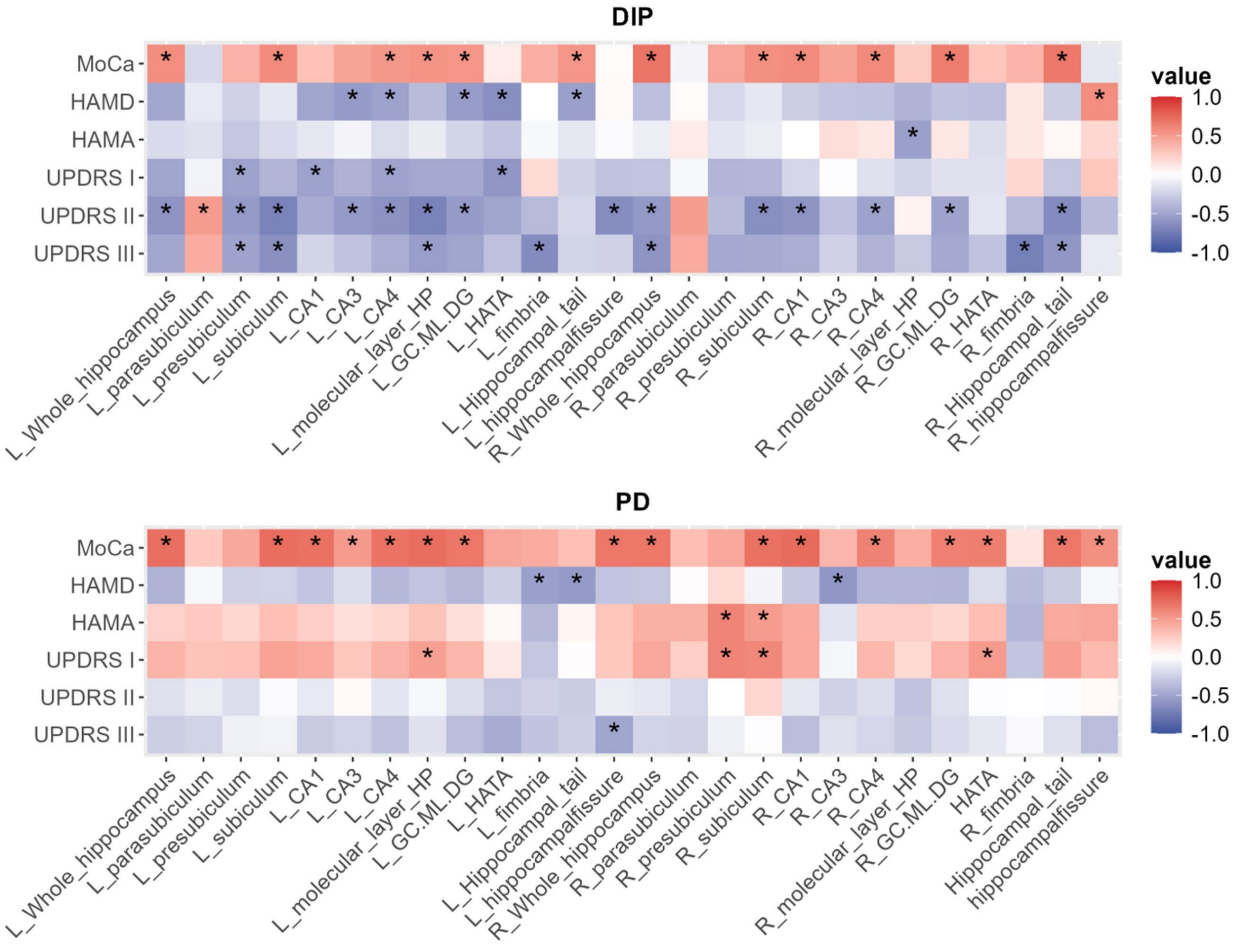
The heatmap of correlations between hippocampal volume parameters and related various clinical measures in DIP and PD group, with *indicating *p* < 0.05.

The results of the correlation analysis are presented in the following heatmap (Figure 3 and Supplementary Tables 1, 2).

In both DIP and PD groups, MoCA scores were positively correlated with bilateral total hippocampal volumes and several subfield volumes, while HAMD scores predominantly exhibited negative correlations with hippocampal volume metrics. According to the heatmap results, the DIP group displayed a greater number of statistically significant correlations between hippocampal volume metrics and UPDRS scores compared to the PD group, with most of these correlations being negative.

### Subgroup analysis based on H-Y staging scale

Patients were grouped using an H-Y stage threshold of grade 2, with those at grade 2 and above combined into a single group. The volumes of the hippocampus and its subfields were compared in both the DIP and PD groups at different H-Y stages, respectively. The results showed no statistically significant differences in the whole hippocampal volume or its subfield volumes between the two groups (Supplementary Tables 3, 4).

## Discussion

This study is the first to investigate hippocampal subfield volume alterations in patients with DIP and their associations with clinical parameters, including cognitive performance (MoCA), emotional states (HAMD), and motor/non-motor symptoms (UPDRS). Our findings revealed significant reductions in hippocampal subfields in the DIP group compared to HCs, revealing a distinct pattern of hippocampal atrophy in DIP. Moreover, the DIP group exhibited more severe hippocampal volume loss than the PD group, which may due to specific mechanistic differences between primary and secondary parkinsonism. These structural changes were significantly correlated with clinical outcomes, particularly cognitive and emotional dysfunction, suggesting that hippocampal atrophy may play a critical role in the course of DIP.

We observed widespread reductions in hippocampal subfields, including the presubiculum, subiculum, GC-ML-DG, molecular_layer_HP, CA1, CA4, hippocampal tail, and fimbria, reflect severe structural damage in DIP, with pronounced atrophy in DIP compared to PD. On one hand, hippocampal alterations are possibly caused by neuronal damage (Fan et al., 2018; Zhu et al., 2018). Our results may suggest that hippocampal atrophy occurs in both DIP and PD, potentially reflecting a common structural vulnerability. Previous researches reported that cognitive impairments in PD have been linked to reductions in hippocampal volume (Xu et al., 2020; Carlesimo et al., 2012). Recent study (Low et al., 2019) found atrophy in the CA1 subfield of the hippocampus in developed PD dementia. Hippocampal volume decrease might serve as a common biomarker of cognitive vulnerability in both degenerative and non-degenerative parkinsonian syndromes.

On the other hand, the more extensive and severe hippocampal atrophy observed in DIP compared to PD when compared to HCs, might reflect unique pathological processes influenced by differences in clinical characteristics. The disease course in DIP is typically shorter than in PD (López-Sendón et al., 2012), and its progression was more quickly (Shiraiwa et al., 2018). This may suggest that hippocampal volume reductions in DIP may be attributed to the acute effects of drug exposure, contrasting with the gradual, progressive neurodegenerative changes observed in PD. Additionally, DIP patients exhibited significantly lower MoCA scores compared to PD patients, indicating more pronounced cognitive impairment, consistent with earlier studies indicating that neurological deficits in DIP are more pronounced than those in PD (Shin and Chung, 2012). We speculated that cognitive vulnerability in DIP may exacerbate hippocampal susceptibility. So, these factors may collectively contribute to the more severe hippocampal atrophy observed in DIP patients.

Cognitive impairment, as reflected by lower MoCA scores in the DIP group, was strongly associated with reduced hippocampal subfield volumes. Specifically, subfields such as the subiculum, CA4, and CA1 showed significant positive correlations with MoCA scores. Firstly, we found the DIP patients exhibited more pronounced cognitive impairment, in addition to the previously mentioned relationship between cognitive impairment and hippocampal changes, cognitive impairment itself has been reported as a significant risk factor in the onset and progression of DIP (López-Sendón et al., 2012). Secondly, a positive correlation was observed between MoCA scores and hippocampal subfield volumes, suggesting that smaller hippocampal volumes were linked to more severe cognitive deficits. Although the causal relationship between these two factors remains unclear, it is possible that the reduction in hippocampal volume could be a result of prolonged cognitive decline, or conversely, that hippocampal atrophy may contribute to the worsening of cognitive function. This underscores the complex interplay between structural brain changes and cognitive performance in DIP, warranting further investigation to elucidate the direction of causality.

The negative correlations between HAMD scores and hippocampal subfield volumes, particularly in the HATA and CA3, suggest that smaller hippocampal volumes are linked to greater depressive symptoms in DIP patients. These regions are crucial for emotional regulation, for example, CA3 is an important subfield involved in depression (Nolan et al., 2020; Roddy et al., 2019). All of them have been reported decreased volume in depression in the majority researches (Nolan et al., 2020; Sun et al., 2023). Moreover, these correlations were stronger in DIP than in PD, indicating that hippocampal atrophy may have a more significant impact on mood disturbances in DIP patients. In addition to the interplay with hippocampal structural changes, gender differences in the occurrence of DIP may also be another contributing factor. Since DIP has a higher prevalence in female (Shin and Chung, 2012), who also have a higher incidence of depression (Eid et al., 2019; Marx et al., 2023), and our DIP sample also included a higher proportion of female participants. So, gender-specific factors may contribute to the more pronounced mood disturbances observed in the DIP group.

While the hippocampus is primarily associated with cognitive and emotional functions, exploring its relationship with motor symptoms may be significant in DIP and PD. Molina et al. (2016) reported that motor impairments in PD patients can predict cognitive deficits in schizophrenia patients, suggesting a potential mediating role of the hippocampus. Ledoux et al. (2014) found that hippocampal dysfunctions can affect motor-related tasks. These results suggested that the hippocampus may play a role in integrating cognitive and motor functions, which could have implications for understanding the pathophysiology of related disorders. In our study, the heatmap analysis revealed distinct patterns of correlation between UPDRS I/II/III and hippocampal subfield volumes in both DIP and PD groups. These findings highlight group-specific differences in how hippocampal subfields relate to motor and non-motor symptoms in DIP and PD. Previous studies have suggested the hippocampus, interacting with the basal ganglia and prefrontal cortex, likely contributed to motor and cognitive integration within the broader cortico-basal ganglia-thalamic circuits (Maurice et al., 2015; Ursino et al., 2020). Notably, the more extensive correlations in the DIP group, particularly with UPDRS-I and III, may suggest a heightened hippocampal involvement in this condition, potentially reflecting its unique pathophysiology. Further research is needed to clarify these associations and to determine whether hippocampal structural changes could serve as biomarkers for symptom severity or progression in DIP and PD.

Additionally, subgroup analysis based on Hoehn and Yahr (H-Y) staging showed no significant differences in hippocampal volumes or subfield volumes between the DIP and PD groups. This lack of significant structural differences may be attributed to the relatively short disease duration in our sample, suggesting that hippocampal structural changes might not yet be detectable.

Although subgroup analysis based on H-Y staging scale did not show significant differences in hippocampal volumes between the DIP and PD groups, we observed significant correlations between hippocampal volumes parameters and UPDRS-III scores. Compared with UPDRS, the H-Y scale can provide a limited evaluation of the severity of motor symptoms (Movement Disorder Society Task Force on Rating Scales for Parkinson's Disease, 2003; Yamada et al., 2022). Another possible explanation is that UPDRS-III, as a continuous measure, offers greater sensitivity in quantifying the severity of motor symptoms. In contrast, the H-Y scale is categorical and provides a general information of disease stage. Furthermore, the relatively small sample sizes within H-Y subgroups may limit the statistical power to detect subtle group differences. Further researches with larger samples and longitudinal follow-up are needed to confirm our findings.

This study has several limitations. Firstly, the relatively small sample size may have reduced the statistical power, potentially affecting the ability to detect minor differences, especially in subgroup analyses. Future studies with larger cohorts are needed to validate these findings. Second, the cross-sectional design precludes causal inferences about the relationship between hippocampal atrophy and clinical outcomes. Longitudinal studies would be valuable to explore how hippocampal changes progress over time and their potential reversibility upon cessation of causative drugs. Third, while the study focused on volumetric changes in hippocampal subfields, functional alterations or connectivity changes were not assessed. Combining structural MRI with functional imaging techniques, such as resting-state fMRI, could provide a more comprehensive understanding of hippocampal dysfunction in DIP. Finally, the heterogeneity of the drugs causing DIP in the recruited population may introduce variability in the observed effects. Stratifying patients by the specific causative agents in future research could clarify drug-specific impacts on hippocampal subfields.

In conclusion, our research provides new perspectives on the structural changes in hippocampal subfields associated with DIP, focusing on significant atrophy in specific regions and their correlations with cognitive, emotional, and motor symptoms. Our findings highlight the need for greater clinical attention to hippocampal alterations in DIP, particularly given its significant impact on cognition, mood, and even motor-related symptoms. Moreover, understanding the unique patterns of hippocampal atrophy in DIP may provide insights into the broader mechanisms underlying drug-induced neurotoxicity and secondary parkinsonism.

## Data Availability

The raw data supporting the conclusions of this article will be made available by the authors, without undue reservation.
