# Supplementary material for: Exploring cognitive and emotional symptoms associated with hippocampal subfield atrophy in drug-induced Parkinsonism
Source: Front Aging Neurosci. 2025 Jul 9;17:1566785. doi: 10.3389/fnagi.2025.1566785 (PMC12283571; doi:10.3389/fnagi.2025.1566785)
Supplement: Supplementary file 1 [file Table_1.docx]

**Supplementary**

**Table 1**  Correlation analysis results in the DIP group

| **r value** | | | | | | |  | **p value** | | | | | | |
| --- | --- | --- | --- | --- | --- | --- | --- | --- | --- | --- | --- | --- | --- | --- |
|  | MoCa | HAMD | HAMA | UPDRS I | UPDRS II | UPDRS III |  |  | MoCa | HAMD | HAMA | UPDRS I | UPDRS II | UPDRS III |
| L_Whole_hippocampus | 0.57 | -0.49 | -0.2 | -0.5 | -0.6 | -0.49 |  | L_Whole_hippocampus | 0.022 | 0.056 | 0.449 | 0.05 | 0.013 | 0.052 |
| L_parasubiculum | -0.21 | -0.12 | -0.17 | -0.07 | 0.5 | 0.42 |  | L_parasubiculum | 0.425 | 0.651 | 0.529 | 0.794 | 0.048 | 0.107 |
| L_presubiculum | 0.39 | -0.25 | -0.31 | -0.52 | -0.56 | -0.51 |  | L_presubiculum | 0.136 | 0.356 | 0.246 | 0.04 | 0.023 | 0.045 |
| L_subiculum | 0.58 | -0.13 | -0.2 | -0.41 | -0.7 | -0.62 |  | L_subiculum | 0.019 | 0.643 | 0.456 | 0.118 | 0.002 | 0.01 |
| L_CA1 | 0.31 | -0.5 | -0.13 | -0.52 | -0.46 | -0.23 |  | L_CA1 | 0.246 | 0.051 | 0.627 | 0.038 | 0.07 | 0.394 |
| L_CA3 | 0.46 | -0.56 | -0.06 | -0.43 | -0.56 | -0.33 |  | L_CA3 | 0.071 | 0.026 | 0.82 | 0.094 | 0.025 | 0.214 |
| L_CA4 | 0.52 | -0.52 | -0.19 | -0.51 | -0.61 | -0.45 |  | L_CA4 | 0.038 | 0.04 | 0.479 | 0.042 | 0.011 | 0.077 |
| L_molecular_layer_HP | 0.54 | -0.37 | -0.11 | -0.48 | -0.7 | -0.54 |  | L_molecular_layer_HP | 0.031 | 0.158 | 0.679 | 0.059 | 0.003 | 0.03 |
| L_ GC-ML-DG | 0.53 | -0.55 | -0.23 | -0.48 | -0.56 | -0.5 |  | L_ GC-ML-DG | 0.033 | 0.029 | 0.394 | 0.057 | 0.025 | 0.05 |
| L_HATA | 0.09 | -0.63 | -0.32 | -0.59 | -0.5 | -0.34 |  | L_HATA | 0.736 | 0.008 | 0.227 | 0.016 | 0.05 | 0.193 |
| L_fimbria | 0.41 | 0 | -0.04 | 0.18 | -0.38 | -0.65 |  | L_fimbria | 0.113 | 0.986 | 0.877 | 0.513 | 0.151 | 0.007 |
| L_Hippocampal_tail | 0.53 | -0.53 | -0.13 | -0.25 | -0.21 | -0.23 |  | L_Hippocampal_tail | 0.035 | 0.036 | 0.637 | 0.359 | 0.426 | 0.382 |
| L_hippocampalfissure | 0.02 | 0.03 | -0.02 | -0.33 | -0.64 | -0.25 |  | L_hippocampalfissure | 0.956 | 0.903 | 0.937 | 0.214 | 0.007 | 0.355 |
| R_Whole_hippocampus | 0.69 | -0.35 | -0.09 | -0.32 | -0.57 | -0.6 |  | R_Whole_hippocampus | 0.003 | 0.184 | 0.751 | 0.23 | 0.02 | 0.015 |
| R_parasubiculum | -0.06 | 0.02 | 0.1 | -0.04 | 0.5 | 0.43 |  | R_parasubiculum | 0.815 | 0.952 | 0.725 | 0.885 | 0.05 | 0.101 |
| R_presubiculum | 0.45 | -0.21 | -0.14 | -0.41 | -0.37 | -0.48 |  | R_presubiculum | 0.082 | 0.427 | 0.613 | 0.111 | 0.155 | 0.059 |
| R_subiculum | 0.56 | -0.13 | -0.1 | -0.4 | -0.63 | -0.47 |  | R_subiculum | 0.025 | 0.625 | 0.722 | 0.129 | 0.008 | 0.064 |
| R_CA1 | 0.58 | -0.27 | 0 | -0.22 | -0.6 | -0.45 |  | R_CA1 | 0.018 | 0.31 | 0.997 | 0.414 | 0.013 | 0.082 |
| R_CA3 | 0.46 | -0.32 | 0.17 | -0.01 | -0.34 | -0.25 |  | R_CA3 | 0.072 | 0.233 | 0.522 | 0.97 | 0.196 | 0.344 |
| R_CA4 | 0.59 | -0.33 | 0.12 | -0.16 | -0.52 | -0.42 |  | R_CA4 | 0.017 | 0.218 | 0.645 | 0.554 | 0.04 | 0.105 |
| R_molecular_layer_HP | 0.26 | -0.42 | -0.53 | -0.23 | 0.06 | -0.29 |  | R_molecular_layer_HP | 0.338 | 0.109 | 0.037 | 0.398 | 0.824 | 0.272 |
| R_GC-ML-DG | 0.65 | -0.33 | 0.12 | -0.16 | -0.51 | -0.48 |  | R_GC-ML-DG | 0.007 | 0.205 | 0.663 | 0.559 | 0.046 | 0.061 |
| R_HATA | 0.29 | -0.35 | -0.18 | -0.16 | -0.14 | -0.33 |  | R_HATA | 0.276 | 0.182 | 0.502 | 0.563 | 0.593 | 0.219 |
| R_fimbria | 0.38 | 0.12 | 0.12 | 0.2 | -0.38 | -0.72 |  | R_fimbria | 0.145 | 0.651 | 0.663 | 0.45 | 0.152 | 0.002 |
| R_Hippocampal_tail | 0.67 | -0.27 | 0.04 | -0.31 | -0.65 | -0.58 |  | R_Hippocampal_tail | 0.005 | 0.308 | 0.897 | 0.237 | 0.007 | 0.019 |
| R_hippocampalfissure | -0.13 | 0.57 | 0.2 | 0.28 | -0.37 | -0.12 |  | R_hippocampalfissure | 0.639 | 0.021 | 0.448 | 0.286 | 0.156 | 0.648 |

**Table 2** Correlation analysis results in the PD group

| **r value** | | | | | | |  | **p value** | | | | | | |
| --- | --- | --- | --- | --- | --- | --- | --- | --- | --- | --- | --- | --- | --- | --- |
|  | MoCa | HAMD | HAMA | UPDRS I | UPDRS II | UPDRS III |  |  | MoCa | HAMD | HAMA | UPDRS I | UPDRS II | UPDRS III |
| L_Whole_hippocampus | 0.72 | -0.42 | 0.23 | 0.38 | -0.17 | -0.27 |  | L_Whole_hippocampus | 0.001 | 0.092 | 0.377 | 0.134 | 0.518 | 0.286 |
| L_parasubiculum | 0.27 | -0.04 | 0.27 | 0.3 | -0.1 | -0.24 |  | L_parasubiculum | 0.288 | 0.875 | 0.288 | 0.25 | 0.69 | 0.356 |
| L_presubiculum | 0.44 | -0.25 | 0.2 | 0.31 | -0.18 | -0.08 |  | L_presubiculum | 0.075 | 0.328 | 0.436 | 0.223 | 0.494 | 0.771 |
| L_subiculum | 0.73 | -0.24 | 0.32 | 0.47 | -0.02 | -0.07 |  | L_subiculum | 0.001 | 0.345 | 0.207 | 0.058 | 0.933 | 0.776 |
| L_CA1 | 0.71 | -0.32 | 0.24 | 0.43 | -0.12 | -0.29 |  | L_CA1 | 0.001 | 0.211 | 0.357 | 0.084 | 0.639 | 0.262 |
| L_CA3 | 0.51 | -0.18 | 0.16 | 0.28 | 0.03 | -0.24 |  | L_CA3 | 0.037 | 0.487 | 0.527 | 0.285 | 0.921 | 0.346 |
| L_CA4 | 0.7 | -0.39 | 0.2 | 0.39 | -0.14 | -0.34 |  | L_CA4 | 0.002 | 0.123 | 0.436 | 0.12 | 0.583 | 0.184 |
| L_molecular_layer_HP | 0.73 | -0.32 | 0.3 | 0.48 | -0.04 | -0.16 |  | L_molecular_layer_HP | 0.001 | 0.207 | 0.239 | 0.049 | 0.87 | 0.54 |
| L_GC-ML-DG | 0.68 | -0.39 | 0.16 | 0.37 | -0.18 | -0.36 |  | L_GC-ML-DG | 0.003 | 0.118 | 0.542 | 0.148 | 0.489 | 0.159 |
| L_HATA | 0.45 | -0.26 | 0.04 | 0.11 | -0.31 | -0.46 |  | L_HATA | 0.07 | 0.316 | 0.88 | 0.672 | 0.233 | 0.064 |
| L_fimbria | 0.42 | -0.53 | -0.39 | -0.31 | -0.25 | -0.33 |  | L_fimbria | 0.089 | 0.028 | 0.117 | 0.226 | 0.327 | 0.201 |
| L_Hippocampal_tail | 0.32 | -0.56 | 0.05 | 0.01 | -0.28 | -0.26 |  | L_Hippocampal_tail | 0.213 | 0.02 | 0.862 | 0.956 | 0.282 | 0.305 |
| L_hippocampalfissure | 0.68 | -0.32 | 0.29 | 0.28 | -0.1 | -0.5 |  | L_hippocampalfissure | 0.003 | 0.213 | 0.251 | 0.27 | 0.694 | 0.039 |
| R_Whole_hippocampus | 0.68 | -0.31 | 0.4 | 0.44 | -0.13 | -0.24 |  | R_Whole_hippocampus | 0.003 | 0.233 | 0.11 | 0.078 | 0.619 | 0.347 |
| R_parasubiculum | 0.33 | 0.01 | 0.4 | 0.25 | -0.22 | -0.25 |  | R_parasubiculum | 0.203 | 0.971 | 0.111 | 0.337 | 0.397 | 0.325 |
| R_presubiculum | 0.44 | 0.18 | 0.62 | 0.62 | 0 | -0.08 |  | R_presubiculum | 0.075 | 0.48 | 0.007 | 0.008 | 0.991 | 0.747 |
| R_subiculum | 0.71 | -0.06 | 0.5 | 0.6 | 0.2 | -0.01 |  | R_subiculum | 0.001 | 0.809 | 0.043 | 0.011 | 0.445 | 0.96 |
| R_CA1 | 0.74 | -0.3 | 0.43 | 0.43 | -0.13 | -0.36 |  | R_CA1 | 0.001 | 0.25 | 0.088 | 0.083 | 0.618 | 0.152 |
| R_CA3 | 0.37 | -0.58 | -0.15 | -0.05 | -0.25 | -0.17 |  | R_CA3 | 0.143 | 0.015 | 0.571 | 0.835 | 0.33 | 0.509 |
| R_CA4 | 0.63 | -0.4 | 0.24 | 0.36 | -0.18 | -0.22 |  | R_CA4 | 0.006 | 0.112 | 0.344 | 0.157 | 0.488 | 0.388 |
| R_molecular_layer_HP | 0.41 | -0.4 | 0.25 | 0.19 | -0.33 | -0.3 |  | R_molecular_layer_HP | 0.105 | 0.113 | 0.332 | 0.472 | 0.193 | 0.247 |
| R_GC-ML-DG | 0.64 | -0.41 | 0.2 | 0.38 | -0.17 | -0.2 |  | R_GC-ML-DG | 0.005 | 0.098 | 0.446 | 0.134 | 0.524 | 0.431 |
| R_HATA | 0.65 | -0.19 | 0.33 | 0.51 | -0.01 | -0.13 |  | R_HATA | 0.004 | 0.474 | 0.192 | 0.038 | 0.956 | 0.616 |
| R_fimbria | 0.13 | -0.37 | -0.4 | -0.32 | 0 | -0.03 |  | R_fimbria | 0.61 | 0.138 | 0.107 | 0.213 | 0.987 | 0.919 |
| R_Hippocampal_tail | 0.68 | -0.28 | 0.43 | 0.48 | -0.01 | -0.17 |  | R_Hippocampal_tail | 0.003 | 0.28 | 0.087 | 0.053 | 0.978 | 0.505 |
| R_hippocampalfissure | 0.56 | -0.04 | 0.45 | 0.35 | 0.03 | -0.36 |  | R_hippocampalfissure | 0.021 | 0.872 | 0.071 | 0.166 | 0.915 | 0.156 |

**Table 3** Comparison of hippocampal volumes across different H-Y stages in DIP groups.

|  | | |  |  |  |  |  |  |
| --- | --- | --- | --- | --- | --- | --- | --- | --- |
|  | Grage 1 (N=6) | |  | Grade ≥ 2 (N=13) | |  | F value | p value |
|  | mean | sd |  | mean | sd |  |  |  |
| L_Whole_hippocampus | 3144.422 | 319.284 |  | 3315.779 | 375.370 |  | 0.899 | 0.359 |
| L_parasubiculum | 60.637 | 10.445 |  | 71.173 | 16.936 |  | 0.759 | 0.398 |
| L_presubiculum | 270.655 | 15.672 |  | 305.257 | 33.376 |  | 6.451 | 0.024 |
| L_subiculum | 370.767 | 24.465 |  | 414.856 | 62.708 |  | 2.597 | 0.129 |
| L_CA1 | 578.307 | 56.004 |  | 593.635 | 63.691 |  | 0.354 | 0.561 |
| L_CA3 | 202.875 | 28.935 |  | 203.550 | 34.228 |  | 0.062 | 0.807 |
| L_CA4 | 223.679 | 28.581 |  | 239.122 | 33.260 |  | 0.915 | 0.355 |
| L_molecular_layer_HP | 495.812 | 51.462 |  | 523.816 | 64.725 |  | 1.145 | 0.303 |
| L_GC-ML-DG | 260.740 | 33.832 |  | 272.542 | 40.604 |  | 0.469 | 0.505 |
| L_HATA | 55.487 | 7.963 |  | 56.546 | 8.892 |  | 0.215 | 0.650 |
| L_fimbria | 62.617 | 12.002 |  | 54.374 | 18.685 |  | 0.251 | 0.624 |
| L_Hippocampal_tail | 562.847 | 111.261 |  | 580.908 | 69.678 |  | 0.057 | 0.814 |
| L_hippocampal-fissure | 136.145 | 30.858 |  | 151.124 | 23.655 |  | 2.599 | 0.129 |
| R_Whole_hippocampus | 3442.779 | 259.115 |  | 3409.467 | 375.455 |  | 0.010 | 0.920 |
| R_parasubiculum | 59.945 | 17.470 |  | 60.856 | 14.805 |  | 0.472 | 0.503 |
| R_presubiculum | 285.213 | 27.734 |  | 287.769 | 35.254 |  | 0.186 | 0.673 |
| R_subiculum | 414.776 | 45.036 |  | 426.660 | 56.269 |  | 1.354 | 0.264 |
| R_CA1 | 622.266 | 45.033 |  | 627.643 | 71.350 |  | 0.003 | 0.954 |
| R_CA3 | 232.779 | 25.269 |  | 226.200 | 35.608 |  | 0.047 | 0.832 |
| R_CA4 | 263.486 | 29.539 |  | 256.297 | 31.988 |  | 0.008 | 0.928 |
| R_molecular_layer_HP | 594.283 | 58.416 |  | 585.511 | 76.290 |  | 0.120 | 0.734 |
| R_GC-ML-DG | 301.572 | 35.132 |  | 287.919 | 39.234 |  | 0.152 | 0.703 |
| R_HATA | 55.671 | 7.079 |  | 50.882 | 10.215 |  | 3.652 | 0.077 |
| R_fimbria | 64.820 | 21.371 |  | 52.933 | 19.213 |  | 0.169 | 0.688 |
| R_Hippocampal_tail | 547.969 | 53.660 |  | 546.798 | 62.474 |  | 0.119 | 0.735 |
| R_hippocampal-fissure | 161.174 | 31.638 |  | 183.131 | 24.632 |  | 4.387 | 0.055 |

**Table 4** Comparison of hippocampal volumes across different H-Y stages in PD groups.

|  | | |  |  |  |  |  |  |
| --- | --- | --- | --- | --- | --- | --- | --- | --- |
|  | Grage 1 (N=12) | |  | Grade ≥2 (N=8) | |  | F value | p value |
|  | mean | sd |  | mean | sd |  |  |  |
| L_Whole_hippocampus | 3490.018 | 242.296 |  | 3479.871 | 557.204 |  | 0.094 | 0.764 |
| L_parasubiculum | 73.865 | 20.144 |  | 73.904 | 26.762 |  | 0.018 | 0.894 |
| L_presubiculum | 330.326 | 44.919 |  | 315.462 | 71.255 |  | 0.003 | 0.954 |
| L_subiculum | 442.802 | 47.649 |  | 451.123 | 100.550 |  | 0.508 | 0.487 |
| L_CA1 | 623.095 | 33.882 |  | 638.441 | 120.387 |  | 0.150 | 0.704 |
| L_CA3 | 206.630 | 12.609 |  | 212.025 | 26.390 |  | 0.281 | 0.604 |
| L_CA4 | 250.574 | 15.651 |  | 251.985 | 35.993 |  | 0.148 | 0.706 |
| L_molecular_layer_HP | 546.115 | 39.041 |  | 557.178 | 93.843 |  | 0.558 | 0.467 |
| L_GC-ML-DG | 284.869 | 22.302 |  | 287.420 | 44.451 |  | 0.137 | 0.716 |
| L_HATA | 58.962 | 9.039 |  | 54.051 | 10.776 |  | 1.209 | 0.289 |
| L_fimbria | 68.528 | 21.446 |  | 63.776 | 17.016 |  | 0.262 | 0.616 |
| L_Hippocampal_tail | 604.252 | 67.012 |  | 574.506 | 80.841 |  | 0.143 | 0.711 |
| L_hippocampal-fissure | 162.240 | 19.786 |  | 162.543 | 32.898 |  | 0.000 | 0.994 |
| R_Whole_hippocampus | 3633.348 | 246.371 |  | 3634.171 | 532.037 |  | 0.515 | 0.484 |
| R_parasubiculum | 74.371 | 20.842 |  | 70.351 | 26.185 |  | 0.003 | 0.957 |
| R_presubiculum | 313.859 | 27.164 |  | 315.550 | 68.819 |  | 0.796 | 0.386 |
| R_subiculum | 447.412 | 45.670 |  | 457.432 | 90.071 |  | 1.498 | 0.240 |
| R_CA1 | 654.969 | 54.861 |  | 659.820 | 87.867 |  | 0.602 | 0.450 |
| R_CA3 | 237.055 | 17.195 |  | 228.304 | 27.871 |  | 0.642 | 0.436 |
| R_CA4 | 267.931 | 17.711 |  | 270.778 | 42.211 |  | 0.293 | 0.596 |
| R_molecular_layer_HP | 632.553 | 77.574 |  | 631.129 | 101.109 |  | 0.069 | 0.796 |
| R_GC-ML-DG | 305.502 | 21.666 |  | 305.782 | 46.143 |  | 0.204 | 0.658 |
| R_HATA | 59.632 | 6.406 |  | 57.539 | 11.283 |  | 1.294 | 0.273 |
| R_fimbria | 68.713 | 18.477 |  | 60.664 | 16.736 |  | 0.009 | 0.924 |
| R_Hippocampal_tail | 571.351 | 47.274 |  | 576.821 | 77.244 |  | 1.140 | 0.303 |
| R_hippocampal-fissure | 172.282 | 33.216 |  | 179.266 | 36.557 |  | 0.292 | 0.597 |
